# Identification of Differentially Expressed Genes Associated with Apple Fruit Ripening and Softening by Suppression Subtractive Hybridization

**DOI:** 10.1371/journal.pone.0146061

**Published:** 2015-12-31

**Authors:** Zongying Zhang, Shenghui Jiang, Nan Wang, Min Li, Xiaohao Ji, Shasha Sun, Jingxuan Liu, Deyun Wang, Haifeng Xu, Sumin Qi, Shujing Wu, Zhangjun Fei, Shouqian Feng, Xuesen Chen

**Affiliations:** 1 State Key Laboratory of Crop Biology, Shandong Agricultural University, Tai’an, Shandong, China; 2 College of Horticulture Sciences, Shandong Agricultural University, Tai’an, Shandong, China; 3 College of Plant Protection, Shandong Agricultural University, Tai’an, Shandong, China; 4 Boyce Thompson Institute for Plant Research, Cornell University, Ithaca, New York, United States of America; National Key Laboratory of Crop Genetic Improvement, CHINA

## Abstract

Apple is one of the most economically important horticultural fruit crops worldwide. It is critical to gain insights into fruit ripening and softening to improve apple fruit quality and extend shelf life. In this study, forward and reverse suppression subtractive hybridization libraries were generated from ‘Taishanzaoxia’ apple fruits sampled around the ethylene climacteric to isolate ripening- and softening-related genes. A set of 648 unigenes were derived from sequence alignment and cluster assembly of 918 expressed sequence tags. According to gene ontology functional classification, 390 out of 443 unigenes (88%) were assigned to the biological process category, 356 unigenes (80%) were classified in the molecular function category, and 381 unigenes (86%) were allocated to the cellular component category. A total of 26 unigenes differentially expressed during fruit development period were analyzed by quantitative RT-PCR. These genes were involved in cell wall modification, anthocyanin biosynthesis, aroma production, stress response, metabolism, transcription, or were non-annotated. Some genes associated with cell wall modification, anthocyanin biosynthesis and aroma production were up-regulated and significantly correlated with ethylene production, suggesting that fruit texture, coloration and aroma may be regulated by ethylene in ‘Taishanzaoxia’. Some of the identified unigenes associated with fruit ripening and softening have not been characterized in public databases. The results contribute to an improved characterization of changes in gene expression during apple fruit ripening and softening.

## Introduction

Apple (*Malus domestica*) is one of the most economically important and widely cultivated horticultural fruit crops worldwide. The fruits are rich in nutrients, non-nutrient components, polyphenols and other phytochemicals beneficial for human health [1]. However, fruit softening represents a major quality problem for apples in the marketplace. Fruit softening is undesirable as it results in decreased shelf life and lower sensory values [2]. The extent of fruit softening varies greatly among cultivars and the maintenance of fruit firmness is an important trait in apple breeding programs. Extensive research on the metabolic processes and molecular mechanisms involved in ethylene production, ethylene signaling pathway, fruit ripening and softening have been carried out, but a full understanding of these processes and the mechanisms responsible in different apple cultivars remains incomplete [3–5].

Ethylene is the dominant trigger for ripening and softening in climacteric fruit [6]. In tomato mutants, fruit ripening and softening are inhibited as a result of abnormal synthesis of ethylene [7, 8]. In apple, fruit softening coincides with the increase of endogenous ethylene production during ripening [3, 6], which can be enhanced by exogenous ethylene treatment and suppressed by inhibitors of ethylene action, such as 1-methylcyclopropene (1-MCP) [4]. There are two committed steps in the biosynthesis of ethylene, namely the formation of aminocyclopropane-1-carboxylic acid (ACC) and its conversion to ethylene, which are encoded by the ACC synthase (ACS) and ACC oxidase (ACO) gene families, respectively [9]. Moreover, *MdACS3a* may determine the ethylene production and shelf life of apple fruits by acting as a switch in the transition between system-1 and system-2 ethylene synthesis [10]. Ethylene biological effects are achieved through genes in the ethylene signaling pathway, including *ethylene receptors* (*ETRs*), *constitutive triple response 1* (*CTR1*), *ethylene insensitive 2* (*EIN2*), *EIN3/EIN3-like* (*EILs*) and *ethylene responsive factors* (*ERFs*) [11, 12]. *ERFs* act by binding to the GCC-box element in promoters of genes responsive to ethylene [13]. In kiwifruit, *AdEIL2* and *AdEIL3* are involved in fruit ripening by activating transcription of *AdACO1* [14]. In tomato, anti-sense *LeERF1* fruits show a longer shelf life, suggesting that *LeERF1* positively modulates fruit ripening and softening [15].

In addition to ethylene and the ethylene signaling pathway, enzymes that modify cell wall pectic and hemi cellulosic polysaccharides are associated with fruit ripening and softening. Differences in the softening rates of ‘Scifresh’ and ‘Royal Gala’ may reflect cell wall structure, which is closely associated with activities of pectin methylesterase (PME) and polygalacturonase (PG) [16]. Fruit ripening and softening are closely associated with *PG* expression level in ‘Golden Delicious’ and ‘Fuji’ apples [17, 18]. Harb et al. suggest that higher expression levels of *PG*, *pectin lyase*, *α-L-arabinofuranosidase* (*AF*), *xyloglucan endotransglycosylase 2 (XET2*) and *expansin 2* (*EXP2*) result in rapid softening of ‘McIntosh’ apples [3]. It is suggested that *AF* and *β-galactosidase* (*β-Gal*) may be more closely associated with shelf life of apple than *PG* and *PME*, especially at the onset of fruit ripening and softening [19]. The above-mentioned results indicate the complexity of the regulatory mechanisms of fruit ripening and softening among different apple cultivars and the necessity for further study.

The present study focused on the apple cultivar ‘Taishanzaoxia’, which is an early-ripening cultivar with excellent fruit appearance and quality characteristics [20]. ‘Taishanzaoxia’ is extremely sensitive to ethylene and fruit firmness decreases rapidly during the late development period coincident with a burst of ethylene production, which hinders its promotion in the apple industry [12, 20]. However, these attributes make the cultivar ideal material for investigation of the mechanisms of fruit ripening and softening, especially the role of ethylene. Li et al. observe that hypersensitive ethylene signaling and *MdPG1* expression lead to fruit ripening and softening in ‘Taishanzaoxia’ [12]. Although 1-MCP treatment significantly suppresses expression of *MdPG1*, fruit firmness still declines but at a slower rate [20]. Other studies of apple show that *PG1*-suppressed fruits are significantly softer than *ACO1*-suppressed fruits [18]. These results suggest that, in addition to *MdPG1*, a suite of enzymes is required for fruit ripening and softening in ‘Taishanzaoxia’.

Subtractive suppression hybridization (SSH) is a method widely used for separation of genes differentially expressed at low levels in two closely related DNA samples [21]. Previous studies have successfully separated differentially expressed genes involved in fruit ripening and softening from apple, banana and Chinese jujube [21–23]. In the present study, forward and reverse SSH libraries were constructed to isolate up- or down-regulated genes involved in fruit ripening and softening in ‘Taishanzaoxia’. The results contribute to improved characterization of the changes in gene expression that occur during fruit ripening and softening in apple. Moreover, elucidation of the regulatory mechanisms of fruit ripening and softening in different apple cultivars is critical for germplasm research, which is beneficial for optimization of fruit quality and fruit breeding programs.

## Materials and Methods

### Plant material and RNA isolation

Fruits of apple ‘Taishanzaoxia’ were obtained from the Shandong Agricultural University fruit breeding orchard (36°26′N, 117°29′E) located in Tai’an, Shandong, China. The fruits were harvested at six development stages (40, 50, 60, 65, 70, 75 days after full bloom) and immediately transferred to the laboratory. Fruits that were free of mechanical injury, insects and diseases were selected for the experiment. The fruits were cut into approximately 1 cm^2^ pieces, frozen in liquid nitrogen and stored at −80°C until use.

For each sample, 2 g of fruit tissue was quickly ground into a fine powder in liquid nitrogen with a mortar. Total RNA was isolated using the RNAprep Pure Kit (TIANGEN, Beijing, China) following the manufacturer’s protocol. One microgram of RNA was run on a 1% agarose gel to check the integrity. The concentration (ng μL^−1^) and quality (A_260_/A_280_) of the total RNA were determined using a Nanodrop 2000 spectrophotometer (Thermo Scientific, Waltham, MA, USA).

### Determination of fruit firmness and ethylene

#### Fruit firmness

The firmness of unpeeled fruit was measured with a TA.XT plus texture analyzer (Stable Microsystems, Godalming, U.K.) with a P/2 columnar probe (2 mm diameter). The test parameter was set as, pre-test speed 2 mm s^-1^, test speed 1 mm s^-1^, post-test speed 10 mm s^-1^, depth of penetration 10 mm, trigger force 10 g. The firmness was automatically obtained by Texture Exponent 32. Each fruit was punctured twice near the equator, and eight replicates were used to test the fruit firmness.

#### Ethylene measurement

Two fruits were sealed in a 1.5 L glass jar and kept at room temperature (24°C) for 6 h. Headspace samples (1 mL) were collected and analyzed with a gas chromatograph (Shimadzu, Kyoto, Japan) equipped with a flame ionization detector. The temperatures of the separation column and detector were 70°C, 120°C respectively; nitrogen and hydrogen were used as the carrier gas at 20 mL min^-1^, 50 mL min^-1^. Ethylene production rate was calculated by peak area quantification. The average ethylene concentration from three jars was calculated and used in further analyses.

### SSH library construction

Two SSH libraries were constructed to isolate differentially expressed genes during fruit ripening and softening. The forward library was constructed to isolate up-regulated genes with samples at 60 days after full bloom (DAFB) as the driver and samples at 70 DAFB as the tester. The reverse library was generated using reciprocal samples to isolate down-regulated genes (S1 Fig).

The SSH libraries were constructed using the PCR-Select^™^ cDNA Subtraction Kit (Clontech, Mountain View, CA, USA), starting with 2 mg mRNA from the tester and driver samples. The mRNA was isolated from total RNA using the FastTrack^®^ MAG mRNA Isolation Kit (Invitrogen, Carlsbad, CA, USA). Differentially expressed cDNA fragments digested with *Rsa*I after a two-round PCR selection were cloned into the pMD18-T vector and transformed into *Escherichia coli* strain DH5a (Invitrogen). Positive clones from LB plates containing 50 mg L^−1^ ampicillin and X-Gal/IPTG were selected for PCR amplification to identify the insert sizes.

PCR amplification was performed to test the subtraction efficiency. The PCR reactions were performed in a total volume of 30 μL and included 22.4 μL sterile water, 3 μL 10× buffer, 1.2 μL of each primer (10 μM), 0.6 μL dNTPs (10 mM), 0.6 μL Taq DNA polymerase (5 U μL^−1^) (Invitrogen) and 1 μL 10-fold diluted subtracted cDNA (2° PCR product) or unsubtracted tester control (2° PCR product). Each PCR was performed as follows: 18 cycles of 94°C for 30 s, 60°C for 30 s, and 68°C for 2 min. Remove 5 μL PCR product from each reaction into a clean tube, and put the rest of the reaction back into the thermal cycler for 5 additional cycles. Repeat the step twice and then examine the 5 μL samples (removed from each reaction after 18, 23, 28 and 33 cycles) on a 1% agarose gel.

### Amplification of cDNA inserts

The primers M13-47 and RV-M were used for PCR amplification of cDNA inserts from white colonies. The PCR reactions were performed in a total volume of 20 μL and included 15.2 μL sterile water, 2 μL 10× buffer, 0.5 μL of each primer (20 μM), 0.5 μL dNTPs (10 mM), 0.3 μL Taq DNA polymerase (5 U μL^−1^) (Invitrogen) and 1 μL bacterial culture. Each PCR was performed as follows: 94°C for 4 min, followed by 30 cycles of 94°C for 30 s, 56°C for 30 s, and 72°C for 3 min, and a final extension at 72°C for 10 min. The PCR products were electrophoresed in 1% agarose gel to confirm the amplification. A subset of positive clones for which PCR products were longer than 100 bp was selected for preparation of plasmid DNA using the Plasmid DNA Extraction Kit (TIANGEN).

### Sequencing and data analysis

The selected positive clones were sequenced with the universal M13 sequencing primer. The raw expressed sequence tag (EST) sequences were generated from sequencing files with the software Phred [24]. The vector, adaptor and low-quality bases were removed from raw ESTs using LUCY [25] and the resulting ESTs were assembled into unigenes using iAssembler [26] with minimum overlap of 40 bp and minimum percent identity of 97. The resulting unigene sequences were compared against the GenBank non-redundant (nr), UniProt (TrEMBL/SwissProt) and Genome Database for Rosaceae. Each unigene was annotated with Gene Ontology (GO) terms and classified into GO slim categories (http://www.geneontology.org/GO.slims.shtml).

### Real-time quantitative RT-PCR analysis

Differential expression analysis of the genes was performed with quantitative real-time RT-PCR (qRT-PCR) in triplicate. First-strand cDNA was synthesized from 1 μg total RNA using the miRcute miRNA First-strand cDNA Synthesis Kit (TIANGEN). The qRT-PCR reactions were performed using a CFX96^™^ Real-Time PCR Detection System (Bio-Rad, Hercules, CA, USA), containing 1 μL cDNA (5-fold dilution), 10 μL TransStart^®^ Top Green qPCR SuperMix (Transgene, Beijing, China), 1 μL of each primer and 7 μL ddH_2_O. The qRT-PCR program was set as 94°C for 30 s, 40 cycles of 94°C for 5 s, 58°C for 15 s and 72°C for 10 s. The *Actin* gene served as an internal control and the relative quantification of specific mRNA levels was performed using the cycle threshold (Ct) 2^−ΔΔCt^ method [27]. Primers used in this study were designed with Primer 6 (S1 Table).

## Results

### Construction of SSH libraries

**Fig 1:**
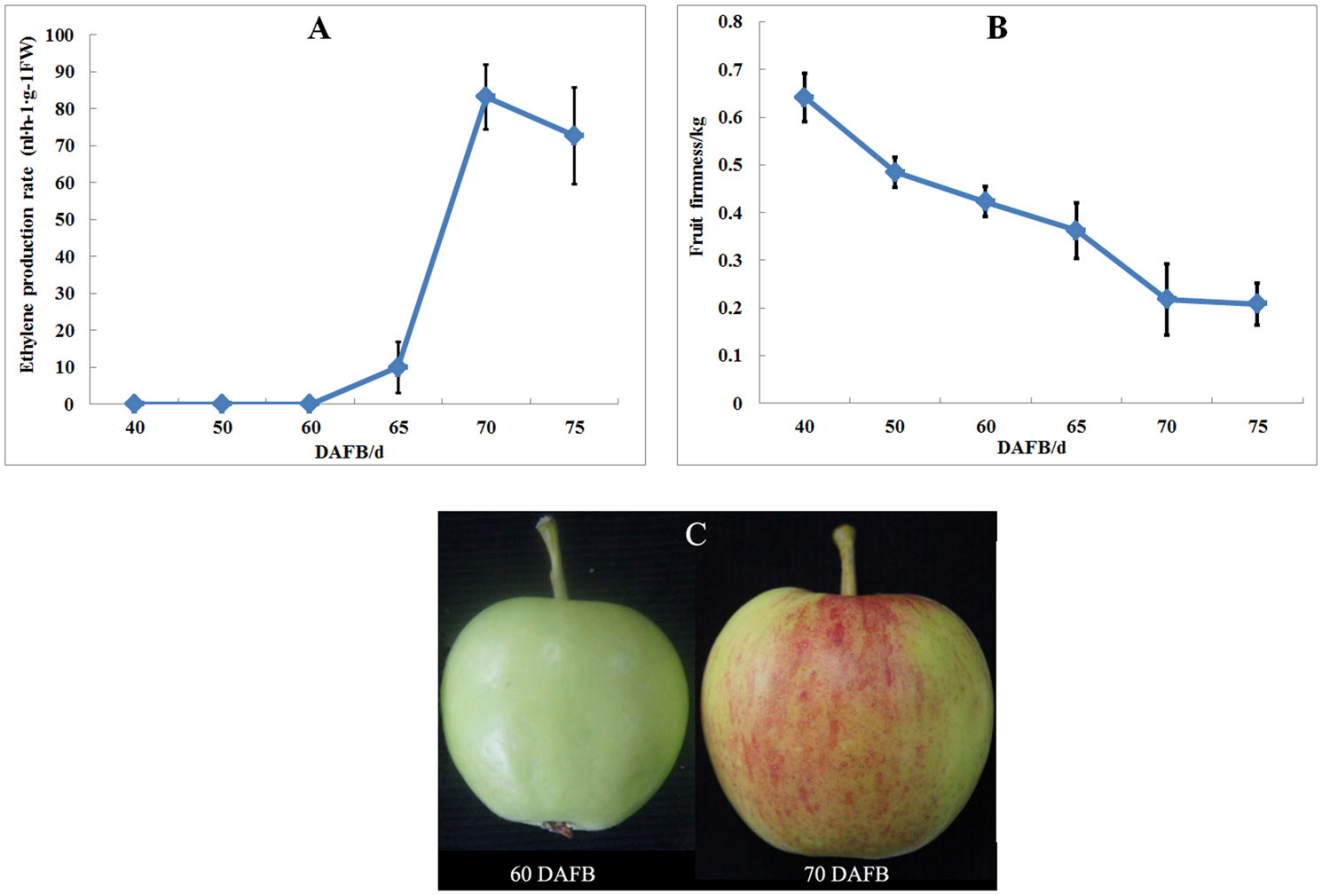
Fruit firmness and ethylene production in apple ‘Taishanzaoxia’ during the fruit development period. (A) Ethylene production rate, (B) Fruit firmness, (C) Samples used for SSH construction.

Ethylene production and fruit firmness were measured during fruit development period to determine sampling time points for construction of SSH libraries. Ethylene production continued to increase during fruit development period while fruit firmness declined continuously (Fig 1). The ethylene biosynthesis burst at 60–70 DAFB accompanied a dramatic decline in fruit firmness. Important gene expression and physiological changes were predicted to occur during this period. Therefore, samples at 60 DAFB and 70 DAFB were used for construction of SSH libraries to identify differentially expressed genes involved in fruit ripening and softening. The subtraction efficiency of *Actin* in two libraries was tested by PCR application, and it was as high as 2^10^-fold, suggesting the subtraction was efficient (S2 Fig). The total number of clones in the forward and reverse libraries was 2600 and 2400, respectively.

### EST sequencing and assembly

**Fig 2:**
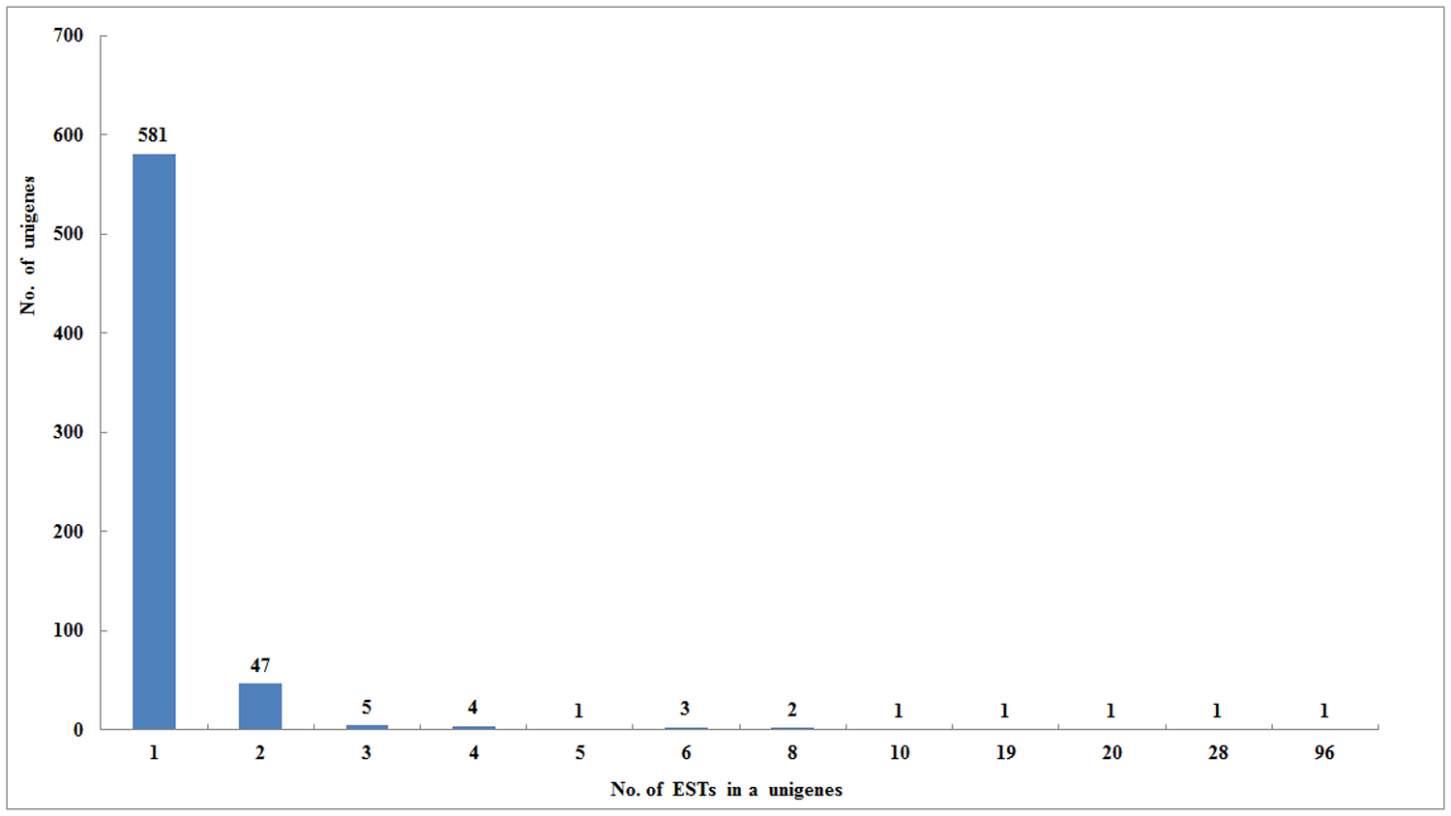
Distribution of the number of EST members in each unigene.

A total of 1032 clones (504 clones in forward library and 528 clones in reverse library) were selected and sequenced, with the positive rate of 89% and 88%, respectively. After removal of low-quality sequences and sequences of bacterial origin, 918 high-quality ESTs ranging between 200 and 1000 bp were obtained, with an average length of 421 bp. These ESTs were further assembled into 648 unigenes, with an average length of 437 bp, among which 67 were contigs (assembled from multiple EST sequences) and 581 were singletons (sequences that could not be assembled into a contig) (Table 1). The number of EST members in unigenes varied from one to 96, with most of the unigenes (581, 90%) present in low copy numbers. Most contigs obtained (70.1%) were composed of two ESTs (Fig 2).

**Table 1:** Summary statistics for the forward and reverse SSH libraries.

Name of library	Tester	Driver	No. of clones	High quality sequence	No. of contigs	No. of singletons	No. of unigenes	Description of transcript clones
Forward library	70 DAFB	60 DAFB	504	451	51	305	356	Up-regulated in flesh tissues
Reverse library	60 DAFB	70 DAFB	528	467	47	276	323	Down-regulated in flesh tissues
Total	—	—	1032	918	67^a^	581	648^a^	Differentially expressed in flesh tissues

Note:

^a^ means 32 contigs were consisted with ESTs from both forward and reverse library.

### Annotation and functional cataloging

**Fig 3:**
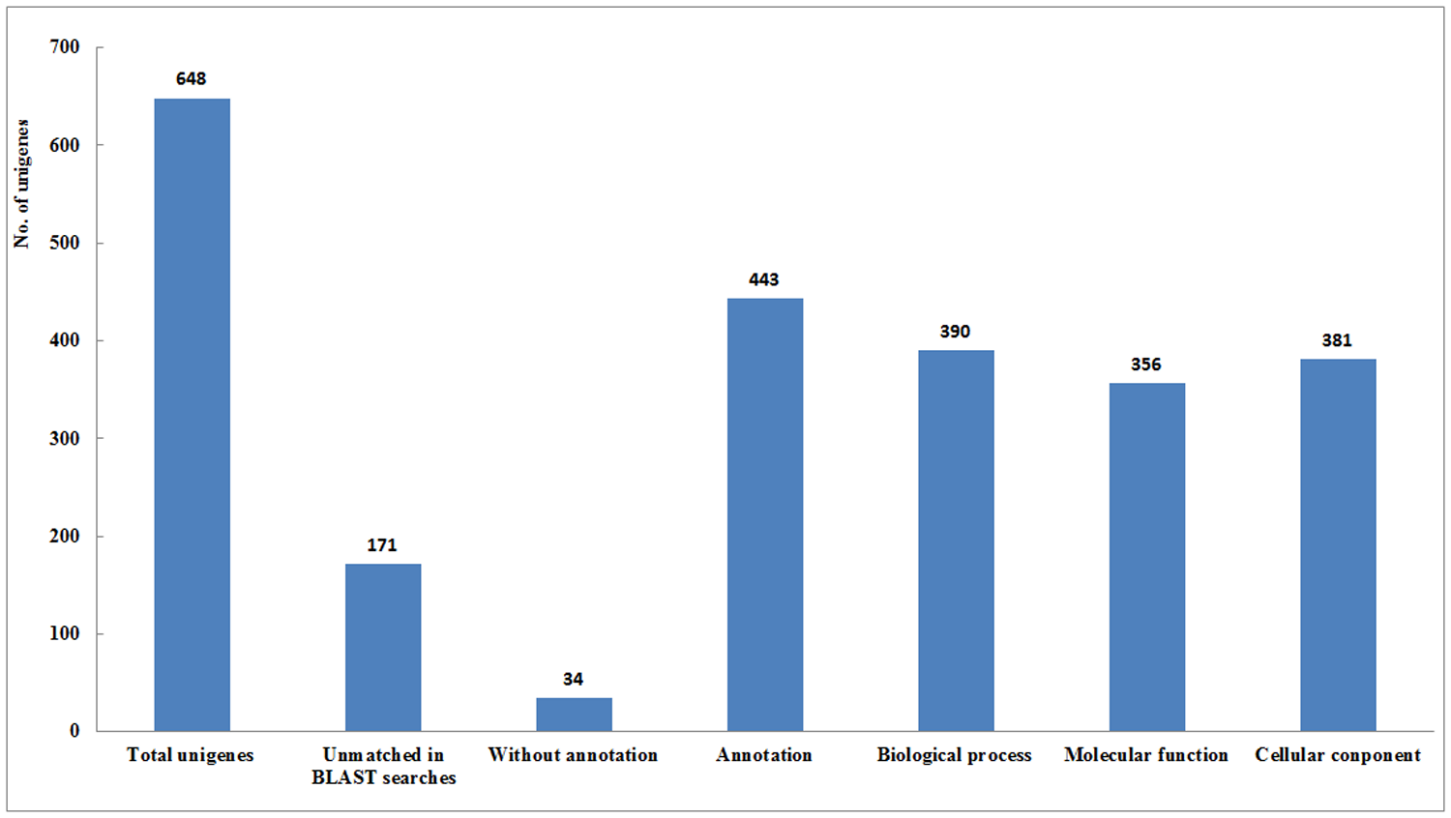
Annotation statistics for unigenes. Unigenes annotated as known proteins with E-value threshold 9E^−6^. Total numbers of unigenes, and unigenes unmatched in BLAST searches, without annotation and with annotation were presented. The unigenes were annotated with GO terms and grouped in three major categories: biological progress, molecular function and cellular component.

The unigenes were further analyzed by searching against the National Center for Biotechnology Information non-redundant (nr) database and Genome Database for Rosaceae using the BLASTX program. A total of 443 (68.4%) unigenes showed significant similarities to known functional gene sequences in the database, and 34 showed matches against genes of unknown function or hypothetical proteins. The remaining 171 sequences did not match with any gene sequence in the database (Fig 3).

The unigenes were further annotated with GO terms and GO slim categories. Based on the GO annotations, the unigenes were classified into three ontology categories and 94 GO slims. A total of 390 unigenes were assigned to 44 GO slims in the biological process category, 356 to 25 GO slims in the molecular function category, and 381 to 25 GO slims in the cellular component category (Fig 3).

**Fig 4:**
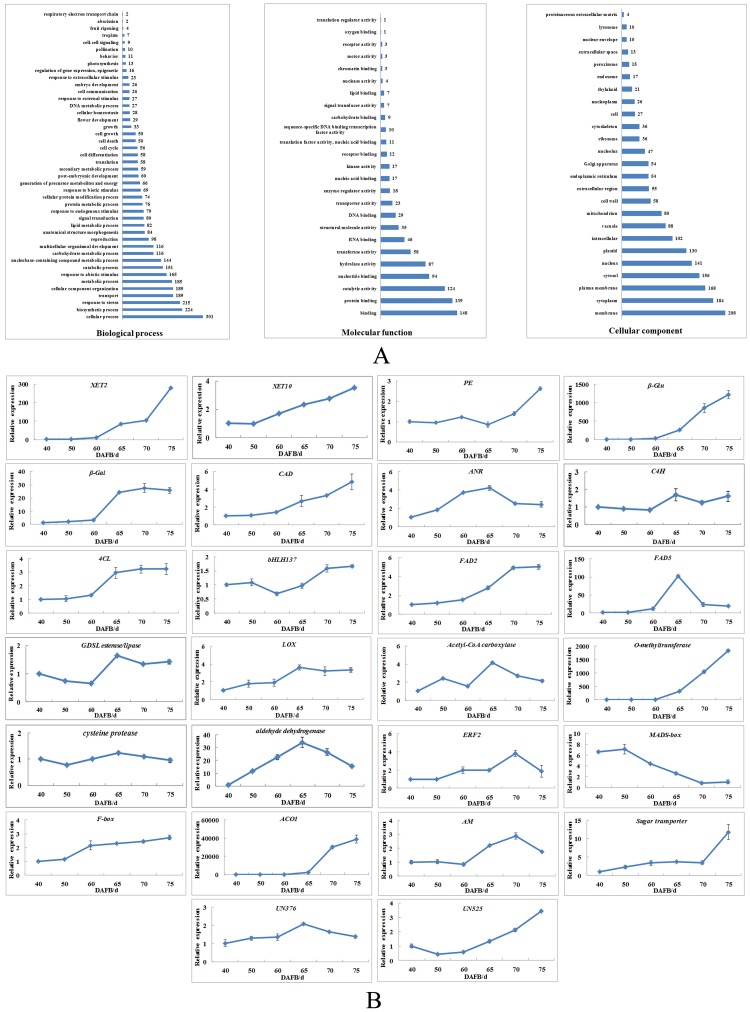
(A) Functional classification of differentially expressed unigenes into GO slim categories. (B) Expression analysis of selected unigenes during the fruit development period. *CAD*: *cinnamyl alcohol dehydrogenase*, *4CL*: *4-coumarate-CoA ligase*, *ANR*: *anthocyanidin reductase-like*, *C4H*: *cinnamic acid hydroxylase*, *FAD2*: *fatty acid desaturase 2*, *FAD5*: *fatty acid desaturase 5*, *LOX*: *lipoxygenase*, *AM*: *β-amylase*.

In the biological process category, ‘cellular process’ was the most prevalent (9% of sequences), followed by ‘biosynthesis process’ (7%) and ‘response to stress’ (6%). In addition, ‘fruit ripening’ (0.1%), regulation of gene expression (0.5%), secondary metabolic process (1.7%) and signal transduction (2.4%) were also identified in the biological process category. In the molecular function category, ‘binding’ was the most prevalent (16%) and ‘hydrolase activity’ (10%), ‘sequence-specific DNA binding transcription factor activity’ (1.1%) and ‘transporter activity’ (2.5%) were also identified. In the cellular component category, ‘membrane’ (12%) was the most dominant term, followed by ‘cell wall’ (3%) (Fig 4A).

### Identification of fruit ripening- and softening-related genes

Although a large amount of sequence information was generated from the SSH libraries in this study, only 68 unigenes were considered to be relevant to fruit ripening and softening on the basis of GO annotations and the higher number of these genes that were sorted into different categories based on their putative functions (Table 2). The largest category, consisting of 13 (19%) genes, was associated with cell wall modification, such as *XET2*, *XET10*, *pectinesterase* (*PE*) and *β-Gal*. Twelve (18%) genes were predicted to be associated with anthocyanin biosynthesis (*4CL*, *ANR* e.g.), aroma production (*FAD*, *LOX* e.g.) and stress response. Among the differentially expressed genes, genes associated with metabolism (*AM* and *ACO1*) and transport accounted for 9% and 6% of the total, respectively. In addition, transcription factors such as *ERF2*, *bHLH137*, *F-box* and *MADS-box* were isolated from the SSH libraries, accounting for 10% of ripening- and softening-related genes.

**Table 2:** Genes differentially expressed during apple fruit ripening and softening.

Unigene No.	Length(bp)	Accession No.	Annotation	Origanism of Best Homology	E-Value	Source
Cell wall modification						
UN061*	746	MDP0000144790	xyloglucan endotransglucosylase/hydrolase 10	Malus x domestica	0	FS
UN088	499	MDP0000320366	probable exocyst complex component 6	Malus x domestica	2E-176	RS
UN203*	378	MDP0000157209	cinnamyl alcohol dehydrogenase	Malus x domestica	3E-115	RS
UN286	404	MDP0000162904	pectinesterase-like	Malus x domestica	7E-98	RS
UN289	509	MDP0000277149	Pectate lyase precursor	Malus x domestica	1E-177	RS
UN313	428	MDP0000408971	annexin D1	Malus x domestica	1E-105	RS
UN357*	350	MDP0000225360	beta-galactosidase	Malus x domestica	8E-45	FS
UN402	459	MDP0000168331	40S ribosomal protein S14	Malus x domestica	2E-101	FS
UN438	421	MDP0000525232	probable protein phosphatase 2C 59	Malus x domestica	6E-83	FS
UN463	479	MDP0000218946	β-1,3-galactosyltransferase 2	Malus x domestica	1E-115	FS
UN469	803	MDP0000866084	β-D-galactosidase	Malus x domestica	0	FS
UN565*	500	MDP0000320017	xyloglucan endotransglycosylase 2	Malus x domestica	2E-139	FS
UN641*	412	MDP0000295562	Glucan endo-1,3-β—glucosidase precursor	Malus x domestica	3E-103	FS
Anthocyanin biosynthesis						
UN011	531	MDP0000195254	snakin-1	Malus x domestica	1E-149	FS
UN015*	834	MDP0000221213	anthocyanidin reductase-like	Malus x domestica	0	RS
UN043	779	MDP0000478473	cytochrome P450 716B2-like	Malus x domestica	0	FS
UN054	880	MDP0000321833	calreticulin-3-like	Malus x domestica	0	FS
UN368	700	MDP0000248148	glucosyltransferase	Malus x domestica	0	FS
UN422	751	MDP0000593031	UDP-glycosyltransferase 87A2-like	Malus x domestica	0	FS
UN459	363	MDP0000371499	isoflavone reductase-like protein 6	Malus x domestica	5E-31	FS
UN481	493	MDP0000219282	glycosyltransferase UGT88A1	Malus x domestica	1E-44	FS
UN499	318	MDP0000523205	naringenin,2-oxoglutarate 3-dioxygenase	Malus x domestica	3E-180	FS
UN504*	393	MDP0000229348	cinnamic acid hydroxylase	Malus x domestica	3E-131	FS
UN511	879	MDP0000568913	anthranilate N-hydroxycinnamoyl/benzoyltransferase	Malus x domestica	0	FS
UN514*	391	MDP0000277093	4-coumarate-CoA ligase	Malus x domestica	2E-27	FS
Aroma production						
UN046*	402	MDP0000284275	fatty acid desaturase 2	Malus x domestica	9E-147	FS
UN114	222	MDP0000806502	4-hydroxyphenylpyruvate dioxygenase	Malus x domestica	1E-104	RS
UN118*	650	MDP0000184619	GDSL esterase/lipase	Malus x domestica	0	RS
UN244*	278	MDP0000753547	lipoxigenase	Malus x domestica	5E-95	FS
UN316*	557	MDP0000233141	acetyl-CoA carboxylase	Malus x domestica	2E-111	RS
UN346	416	MDP0000412263	probable ribose-5-phosphate isomerase	Malus x domestica	5E-62	FS
UN393	557	MDP0000169311	linoleate 13S-lipoxygenase 2–1	Malus x domestica	7E-136	FS
UN414	443	MDP0000295806	aspartate aminotransferase 2	Malus x domestica	3E-103	FS
UN474*	496	MDP0000279839	O-methyltransferase	Malus x domestica	2E-151	FS
UN510*	231	MDP0000129670	fatty acid desaturase 5	Malus x domestica	8E-07	FS
UN536	522	MDP0000298646	cytochrome P450	Malus x domestica	0	FS
UN599	822	XM_008369286.1	DNA-(apurinic or apyrimidinic site) lyase	Malus x domestica	0	FS
Stress response						
UN029	410	MDP0000867730	high molecular weight heat shock protein	Malus x domestica	7E-21	FS
UN044	821	MDP0000207137	glutathione peroxidase 8	Malus x domestica	0	RS
UN055	468	MDP0000128468	abscisic stress ripening protein	Malus x domestica	4E-69	RS
UN112*	307	MDP0000764121	cysteine protease	Malus x domestica	9E-174	RS
UN137	363	MDP0000198482	glyceraldehyde-3-phosphate dehydrogenase	Malus x domestica	4E-53	RS
UN180	346	MDP0000150587	putative aconitase	Malus x domestica	0	RS
UN254	520	MDP0000625137	protein disulfide isomerase	Malus x domestica	3E-150	RS
UN403*	479	MDP0000260947	aldehyde dehydrogenase	Malus x domestica	0	FS
UN523	434	MDP0000413395	NADH-cytochrome b5 reductase-like protein	Malus x domestica	2E-104	FS
UN567	533	MDP0000120022	Acid phosphatase 1 precursor	Malus x domestica	1E-180	FS
UN586	412	MDP0000519575	peroxiredoxin	Malus x domestica	0	FS
UN609	798	MDP0000239328	methylmalonate-semialdehyde dehydrogenase	Malus x domestica	0	FS
Transcription						
UN041	584	MDP0000295589	ARF domain class transcription factor	Malus x domestica	0	FS
UN212	410	MDP0000534977	TCP domain class transcription factor	Malus x domestica	1E-133	RS
UN386	856	MDP0000262032	NAC domain class transcription factor	Malus x domestica	0	FS
UN490*	444	MDP0000517257	ethylene response factor 2	Malus x domestica	9E-70	FS
UN496*	666	MDP0000013331	MADS-box protein	Malus x domestica	0	FS
UN575	521	MDP0000269701	DNA-directed RNA polymerase subunit	Malus x domestica	6E-176	FS
UN596*	482	MDP0000261131	F-box family protein	Malus x domestica	6E-142	FS
UN600*	493	MDP0000410728	transcription factor bHLH137	Malus x domestica	2E-58	FS
Metabolism						
UN018*	326	MDP0000195885	1-aminocyclopropane-1-carboxylate oxidase 1	Malus x domestica	3E-158	FS
UN030	541	MDP0000793077	cobalamine-independent methionine synthase	Malus x domestica	5E-115	FS
UN070	816	MDP0000661864	phosphoenolpyruvate carboxylase kinase	Malus x domestica	0	RS
UN166	514	MDP0000293776	phosphofructokinase beta subunit	Malus x domestica	0	RS
UN437*	490	MDP0000196961	β-amylase	Malus x domestica	1E-140	FS
UN542	284	MDP0000221561	malic enzyme	Malus x domestica	2E-153	FS
Transport						
UN199*	325	MDP0000281884	sugar transporter	Malus x domestica	2E-23	RS
UN302	695	MDP0000312731	thioredoxin domain-containing protein 9	Malus x domestica	0	RS
UN306	570	MDP0000767304	ADP-ribosylation factor	Malus x domestica	0	RS
UN309	499	MDP0000181026	Endoplasmic reticulum vesicle transporter protein	Malus x domestica	5E-47	RS
Not annotated						
UN376*	414	MDP0000223932	hypothetical protein	Malus x domestica	2E-144	FS
UN525*	400	MDP0000233440	uncharacterized protein	Malus x domestica	6E-86	FS

Note:

* means unigenes chosen for qRT-PCR analysis.

### Analysis of differential expression by qRT-PCR

To validate the SSH data, 26 differentially expressed unigenes associated with cell wall modification, anthocyanin biosynthesis, aroma production, stress response, transcription, metabolism, transport, and non-annotated unigenes were analyzed by qRT-PCR (Fig 4B). Most genes (25, 96%) were up-regulated during fruit development period. Only one *MADS-box* gene showed a down-regulated expression pattern, which accounted for 80% of the total expression during 40–60 DAFB and was negatively correlated with ethylene production. The expression of genes associated with cell wall modification (*XET2*, *XET10*, *β-Glu*, *β-Gal* and *CAD*), anthocyanin biosynthesis (*4CL* and *bHLH*) and aroma production (*FAD2* and *O-methyltransferase*) was significantly up-regulated during 60–70 DAFB and showed a significant positive correlation with ethylene production. Moreover, the expression of *ACO1*, *ERF2* and *UN525* was also significantly correlated with ethylene production (Table 3). Although it was not significantly correlated with ethylene production, they were all up-regulated during 60–70 DAFB, such as *LOX*, *AM* and *GDSL esterase/lipase et al* (Fig 4B).

**Table 3:** Gene expression statistics during the fruit development period.

Gene name	Soure	$\frac{\text{The expression during 40}-\text{60 DAFB}}{\text{The total expression during 40}-\text{75 DAFB}}(\%)$	$\frac{\text{The expression during 65}-\text{75 DAFB}}{\text{The total expression during 40}-\text{75 DAFB}}(\%)$	Correlation with ethylene
*XET2*	FS	3	97	0.780*
*XET10*	FS	30	70	0.898*
*PE*	RS	40	60	0.710
*β-Glu*	FS	2	98	0.949**
*β-Gal*	FS	7	93	0.812*
*CAD*	RS	24	76	0.863*
*4CL*	FS	26	74	0.822*
*ANR*	RS	42	58	-0.026
*C4H*	FS	37	63	0.511
*bHLH137*	FS	40	60	0.921**
*FAD2*	FS	22	78	0.962**
*FAD5*	FS	10	90	-0.008
*acetyl-CoA carboxylase*	RS	36	64	0.172
*LOX*	FS	32	68	0.654
*O-methyltransferase*	FS	0	100	0.909*
*GDSL esterase/lipase*	RS	35	65	0.552
*cysteine protease*	RS	46	54	0.151
*aldehyde dehydrogenase*	FS	32	68	0.258
*AM*	FS	30	70	0.757
*ACO1*	FS	0	100	0.917**
*ERF2*	FS	34	66	0.753
*MADS-box*	FS	80	20	-0.849*
*F-box*	FS	39	61	0.702
*sugar transporter*	RS	26	74	0.632
*UN376*	FS	42	58	0.198
*UN525*	FS	23	77	0.869*

Note:

* means the significant level of 5% and

** means the significant level of 1%.

## Discussion

Crispness and a sweet-tart flavor in apples are sought-after consumer traits, with texture being a principal quality attribute [3]. Cultivars such as ‘Fuji’ are widely cultivated around the world because of their excellent fresh quality and longer shelf life. A burst of ethylene production occurs during late development period of ‘Taishanzaoxia’ fruit, and 1-MCP treatment significantly inhibits ethylene production, which indicates that ‘Taishanzaoxia’ is extremely sensitive to ethylene [12, 20]. Based on previous studies, we propose that apple cultivars can be divided into three types according to sensitivity to ethylene: ethylene-insensitive (‘Fuji’ e.g.), ethylene-intermediate (‘Golden Delicious’ e.g.) and ethylene-sensitive (‘Taishanzaoxia’, ‘Jonagold’ e.g.) [28]. The burst of ethylene production has negative effects on fruit quality but provides a suitable model for investigation of fruit ripening and softening processes dependent on ethylene.

### Ethylene biosynthesis and signal transduction

*MdACS1* is linked to fruit internal ethylene concentration (IEC) with the *MdACS1-1* allele linked to high IEC [5]. We found that the allelotype of ‘Taishanzaoxia’ was *MdACS1-1/-1*, which was consistent with elevated expression of *MdACS1* and ethylene burst during the late development period (data not shown). Previous studies have shown that *MdACO1* independently affects the internal ethylene concentration and *MdACO1*-suppressed apple fruits produce no detectable increase in endogenous ethylene concentration [6, 29]. *ERFs* represent the last step in the ethylene signaling pathway, and the increased expression of *MdERF2* in ripening fruits is repressed by 1-MCP treatment [30]. In this study, *ACO1* and *ERF2* were isolated from the forward library and were up-regulated during fruit development period and significantly correlated with ethylene production (Fig 4B, Table 3). Consistent with the present results, the elevated expression of *ACO1* and *ERF2* is accompanied by increased ethylene production, which suggests that both *ACO1* and *ERF2* play an important role in regulation of fruit ripening and softening through ethylene biosynthesis and signal transduction.

### Cell wall modification

Fruit softening is probably caused by the cumulative effect of a range of modifications occurring in the network of polymers that make up the primary cell wall [31]. In the present study, six unigenes associated with cell wall modification were identified, namely *XET2*, *XET10*, *PE*, *β-Glu*, *β-Gal* and *CAD*. XET is thought to play a key role in fruit softening through disassembly of xyloglucan and preparation for further modification by other enzymes [32]. PE catalyzes the hydrolytic de-esterification of pectins causing pectic chain esterification, which is further hydrolyzed to pectate by polygalacturonase [33]. β-Glu is likely to target the glucan backbone of xyloglucan, the hemicellulosic polysaccharide that bridges cellulose microfibrils [34]. A decrease in the galactose content of the cell wall is associated with an increase of β-Gal activity during fruit ripening [35]. CAD is a key enzyme involved in lignin biosynthesis, which is an important component of secondary cell walls and provides essential strength, hydrophobicity and resistance to the harsh external environment to the cell wall [36]. Expression of the five genes (*XET2*, *XET10*, *β-Glu*, *β-Gal* and *CAD*) was significantly correlated with fruit firmness and ethylene production, suggesting that fruit softening was closely associated with elevated expression of these ethylene-regulated genes. In the soft/crisp strains of *Malus sieversii* f. *niedzwetzkyana*, fruit softening in the early and late development periods may result from differential expression of *XET* gene family members dependent on or independent of ethylene [37]. Previous studies show that the transcripts of *XET2* and *XET10* are the most abundant in ripe fruits [29], which is consistent with the current results. However, the roles of other *XET* family members dependent on or independent of ethylene in fruit ripening and softening are unknown. Therefore, an important focus of future research will be to determine the roles of ethylene or other upstream regulators in regulating *XET* family members and other cell-wall-modifying genes during fruit ripening and softening.

### Anthocyanin biosynthesis

In this study, SSH libraries were constructed to identify differentially expressed genes involved in fruit ripening and softening, especially softening-related genes. Interestingly, genes associated with anthocyanin biosynthesis and aroma production were identified from the libraries. Anthocyanins are water-soluble flavonoid pigments that are important contributors of the red skin coloration of apple fruits [38]. The regulation of anthocyanin biosynthesis is mainly at the level of transcriptional regulation of structural genes by transcription factors [39]. The transcription factors that control anthocyanin biosynthesis include *MYB*, *bHLH* and *WD40* [40]. We isolated a novel transcription factor, *bHLH137*, from the forward library. In addition, structural genes such as *C4H* and *4CL* were also identified from the forward library. These genes showed an up-regulated expression pattern, which was consistent with their positively regulatory roles in the anthocyanin biosynthesis pathway. In the reverse library, *ANR* was observed to be down-regulated during 60–70 DAFB. *ANR* catalyzes reduction of anthocyanidins to form flavan-3-ols, thus the present findings are consistent with its negative role in the anthocyanin biosynthesis pathway [38]. Correlation analysis showed that only *4CL* and *bHLH137* were significantly correlated with ethylene concentration, which suggested that ethylene played an important role in anthocyanin biosynthesis in ‘Taishanzaoxia’. This observation verified the results of a previous study that accumulation of anthocyanidin was dependent on ethylene in ‘Taishanzaoxia’ [41]. Although *MYB* is thought to play an important role in anthocyanin biosynthesis, it was not isolated from the present libraries. *MYB* may be involved in anthocyanidin biosynthesis through other regulatory pathways, such as light and temperature [42]. Efficient induction of anthocyanin biosynthesis in transient assays by *MdMYB10* is dependent on the co-expression of two distinct *bHLH* proteins from apple, *MdbHLH3* and *MdbHLH33* [43]. Therefore, further investigations are needed to explain the interaction of *bHLH137* with *MYB* and the role of *bHLH137* in regulating the expression of *C4H*, *4CL* and *ANR*.

### Aroma production

In apple, the typical aroma compounds are fruity esters that develop during ripening with the maximum endogenous ester concentration occurring at the climacteric peak [44]. These compounds can be broadly separated into straight- and branched-chain esters. Straight-chain esters are synthesized from fatty acids via the lipoxygenase (LOX) pathway, whereas branched-chain esters are produced from the metabolism of branched-chain amino acids such as isoleucine [45]. Fatty acids are important precursors in the formation of the characteristic aroma in tomato, apple, kiwifruit and pear [46]. *FAD* is involved in biosynthesis of straight-chain esters by mediating accumulation of linoleic acid and linolenic acid as substrates [47]. Linoleic acid and linolenic acid are converted to hydroperoxide fatty acids via LOX activity [48]. *Acetyl CoA*, *FAD2*, *FAD5* and *LOX* were isolated from the SSH libraries and the elevated expression of these genes was consistent with the maximum concentration of esters during the late fruit development period in ‘Taishanzaoxia’ [49]. In addition, *GDSL esterase/lipas*e and *O-methyltransferase* were also identified from the libraries. *FAD2* and *O-methyltransferase* were up-regulated and significantly correlated with ethylene production. The identification of these aroma-related genes supported the previous hypothesis that aroma production may be regulated by ethylene in ‘Taishanzaoxia’ [50].

### Stress response

Fruit ripening and softening have been considered a form of stress for fruits [23]. A suite of unigenes coding for stress-related proteins was identified from the SSH libraries, such as *cysteine protease* and *aldehyde dehydrogenase*. The *cysteine protease* is involved in apoptosis, biotic and abiotic stress, whereas *aldehyde dehydrogenase* is involved in acetaldehyde detoxification, participation in intermediary metabolism, protection from osmotic stress and generation of NAD(P)H [51, 52]. The elevated expression of these genes verified that they may be involved in fruit ripening and softening as a stress response.

### Metabolism, transport and transcription

In addition, *AM* and *sugar transporter* also showed an increasing expression pattern during 40–75 DAFB, suggesting that both genes may play an important role in fruit ripening and softening. In addition, we identified unigenes similar to *F-box* and *MADS-box*. In Arabidopsis, *F-box* proteins direct the ubiquitination and subsequent degradation of positive regulators of ethylene action by incorporation into SCF E3 complexes and interaction with *EIN3/EIL* transcription factors [53]. *F-box* may be involved in fruit ripening and softening by regulation of the ethylene signaling pathway. *MADS-box* is thought to play an important role in regulation of fruit ripening and softening because *MADS-RIN*, a member of the *MADS-box* family of transcription regulators, is an essential regulator of fruit ripening, acting as an upstream regulatory cascade of ethylene, which interacts with promoters of genes involved in fruit ripening and softening, such as *ACS*, *PG2A*, *EXP1*, ethylene receptor *NR*, *E4* and *E8* [54]. Interestingly, *MADS-box* was down-regulated and showed a significantly negative correlation with ethylene, and thus may be a negative regulator in initiating the ethylene burst. It would be of interest in future studies to determine the roles of *F-box* and *MADS-box* interaction with ethylene signaling elements and ripening- and softening-related genes.

This study also identified a large number of genes did not match any sequences currently available in public databases or show homologies to known sequences with unknown functions, such as *UN376* and *UN525*, suggesting that the mechanism of fruit ripening and softening is complex and much remains unknown. Overall, the present study provides valuable information on the isolation and monitoring of genes differentially expressed at lower levels during apple fruit ripening and softening, and especially indicates that fruit texture, coloration and aroma may be regulated by ethylene in ‘Taishanzaoxia’. The information is a valuable foundation for subsequent research to elucidate the ethylene-dependent regulatory network of fruit texture, coloration and aroma to gain a better understanding of biochemical events that occur during fruit ripening and softening.

## Conclusions

We identified differentially expressed genes involved in fruit ripening and softening in ‘Taishanzaoxia’ apple. Twenty-six genes associated with cell wall modification, anthocyanin biosynthesis, aroma production, stress response, metabolism, and transcription as well as non-annotated genes are indicated to play important roles in fruit ripening and softening. ‘Taishanzaoxia’ apple is extremely sensitive to ethylene and fruit texture, coloration and aroma may be all regulated by ethylene. The present findings provide novel insights into the ethylene-dependent regulatory mechanism of fruit ripening and softening, and further enrich our knowledge of the factors that contribute to fruit quality.

## Supporting Information

S1 Fig Flow chart summarizing the construction of SSH libraries and data analysis procedures. (TIF)

S2 Fig Analysis of subtraction efficiency by PCR amplification. (TIF)

S1 Table Primers used for qRT-PCR. (XLSX)

S2 Table Unigene Sequences. (XLSX)
