# Supplementary figures and images for: Identification of Differentially Expressed Genes Associated with Apple Fruit Ripening and Softening by Suppression Subtractive Hybridization
Source: PLoS One. 2015 Dec 31;10(12):e0146061. doi: 10.1371/journal.pone.0146061 (PMC4697835; doi:10.1371/journal.pone.0146061)

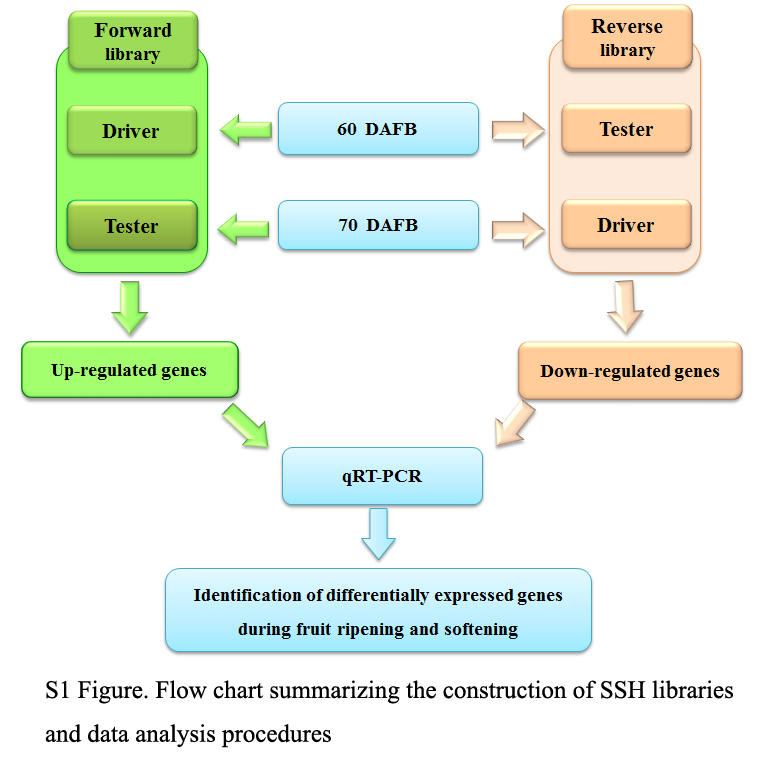

Supplement: S1 Fig — (TIF) [file pone.0146061.s001.tif]

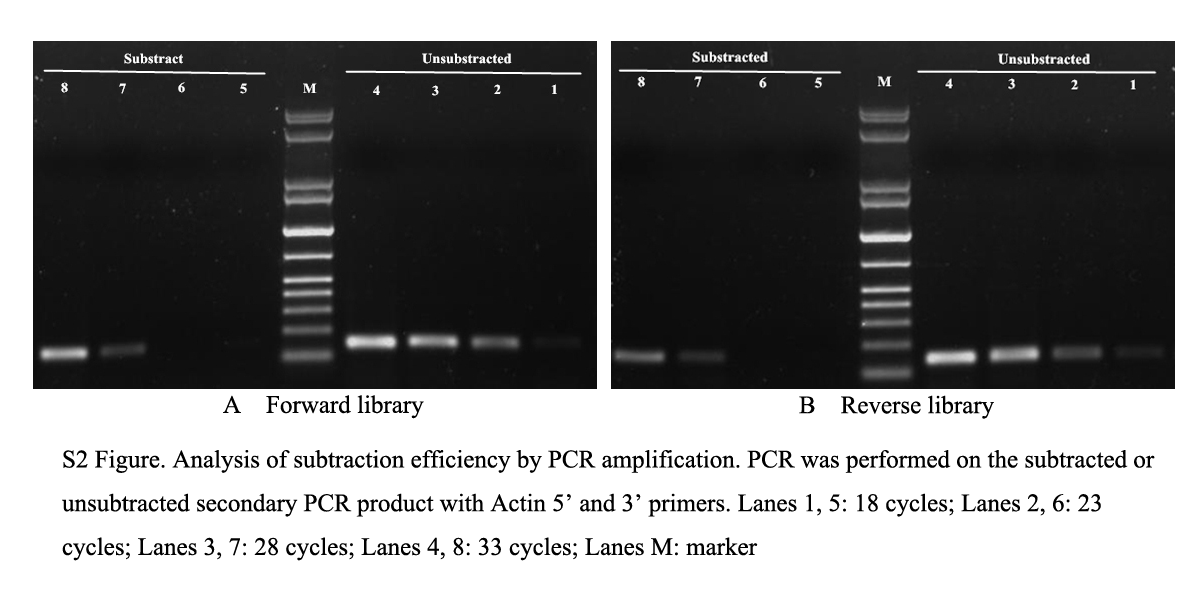

Supplement: S2 Fig — (TIF) [file pone.0146061.s002.tif]
